# Upregulation of Ferroptosis-Related Fanconi Anemia Group D2 is a Poor Prognostic Factor and an Indicator of Tumor Immune Cell Infiltration in Lung Adenocarcinoma

**DOI:** 10.3389/fgene.2022.825685

**Published:** 2022-05-11

**Authors:** Jingtao Zhang, Dongli Wang, Xiubao Chen, Lingyun Ji, Minmin Yu, Minghao Guo, Dexin Zhang, Weida Chen, Fei Xu

**Affiliations:** ^1^ College of Traditional Chinese Medicine, Shandong University of Traditional Chinese Medicine, Jinan, China; ^2^ Digestive Department, Affiliated Hospital of Shandong University of Traditional Chinese Medicine, Jinan, China; ^3^ Department of Geriatric Medicine, Affiliated Hospital of Shandong University of Traditional Chinese Medicine, Jinan, China; ^4^ First Clinical Medical College, Shandong University of Traditional Chinese Medicine, Jinan, China; ^5^ Department of Pathology, Affiliated Hospital of Shandong University of Traditional Chinese Medicine, Jinan, China

**Keywords:** FANCD2, lung adenocarcinoma, ferroptosis, tumor-infiltrating immune cells, biomarker, prognosis

## Abstract

Fanconi anemia (FA) group D2 (*FANCD2*) is a ferroptosis-related gene crucial for DNA damage repair and negative ferroptosis regulation. Our study aimed to evaluate its prognostic value as well as its association with ferroptosis and immune infiltration in lung adenocarcinoma (LUAD). Transcriptome sequencing data, clinical information, and immunohistochemistry data were collected from the TCGA, GEO, and HPA databases, respectively, for three independent cohorts. Univariate and multivariate analyses were used to assess the correlations between FANCD2 expression and overall survival or clinicopathological parameters. cBioPortal was utilized to investigate the *FANCD2* alteration status. Gene and protein networks based on FANCD2 interactions were generated using GeneMANIA and STRING, respectively. Based on the CancerSEA database, the function of FANCD2 was explored at the single-cell level. The relationships between *FANCD2* expression levels and tumor-infiltrating immune cells and their equivalent gene signatures were analyzed using TIMER, GEPIA, TISIDB, and ssGSEA databases. CIBERSORT was used to analyze the relevance of the infiltration of 24 types of immune cells. The results revealed that FANCD2 expression was significantly upregulated in LUAD and lung squamous cell carcinoma (LUSC) tissues than that in normal tissues. Further, the overexpression of FANCD2 was closely associated with poor survival for Patients with LUAD but not for patients with LUSC. FANCD2 expression levels were related to tumor-infiltrating immune cells and their matching gene signatures, including CD8^+^ T cells, natural killer (NK) cells, dendritic cells (DC), and Th2 cells in cases of LUAD. Therefore, FANCD2 was identified as a crucial molecule underlying the synergistic effects of ferroptosis and immunotherapy for Patients with LUAD.

## Introduction

In the past decades, lung cancer has remained the major contributing factor to cancer-related deaths worldwide. According to available data, 2.2 million new cases of lung cancer are diagnosed each year, with 1.8 million people dying from the disease annually (Sung et al., 2021). Non-small cell lung cancer (NSCLC), which includes lung adenocarcinoma (LUAD) and lung squamous cell carcinoma (LUSC), contributes to approximately 85% of all lung cancer cases. However, the majority of patients are in an advanced, unresectable stage of the disease at the time of diagnosis (Brahmer et al., 2018), which is associated with a low overall median 5-year survival rate (Sung et al., 2021). The incidence and mortality of LUAD are increasing; it has now surpassed squamous cell carcinoma to become the most common histological subtype of NSCLC (Jemal et al., 2011; Siegel et al., 2017). Despite the active treatment measures available for LUAD, it has the highest mortality rate among all cancers; this might be associated with its tendency to metastasize at an early stage (Deng et al., 2019; Zhang D. et al., 2019; Li et al., 2021). Thus, it is of critical importance to develop novel and more effective therapeutic strategies for LUAD. For this, it is vital to more intensively probe the molecular pathology of the disease.

Ferroptosis is an iron-dependent form of regulated cell death induced by iron-dependent lipid peroxidation owing to metabolic dysfunction; it is distinct from apoptosis, cell necrosis, and autophagy (Stockwell et al., 2017; Gao and Jiang, 2018). The ferroptosis pathway can restrain tumor growth and induce cancer cell death. Its induction has thus received widespread attention as a potential novel anti-tumor treatment strategy (Hassannia et al., 2019; Liang et al., 2019; Bebber et al., 2020). Inducing ferroptosis has been reported to suppress LUAD by regulating lipid peroxidation to promote tumor cell death (Ma et al., 2021; Wang et al., 2021; Zhang Y. et al., 2021). In addition, ferroptosis is also connected to cell immunity and may have applications in cancer immunotherapy (Wang et al., 2019).

With the rapid advances in high-throughput sequencing technologies and transcriptome sequencing [RNA sequencing (RNA-seq)], an increasing number of key driver oncogenes are being discovered. However, it is necessary to identify additional key driver genes, particularly those affecting the tumor immune microenvironment (TIME) in LUAD. The Fanconi anemia (FA) pathway plays an important role in DNA damage repair by blocking DNA replication, for instance, *via* interstrand cross-links. FANCD2, a member of the FA family of proteins, forms a FANCD2-FANCI heterodimer with FANCI and participates in DNA damage repair via the FA pathway (Nalepa and Clapp, 2018). Some studies have demonstrated that FANCD2 depletion enhances interstrand crosslink (ICL) agent-induced DNA damage sensitivity and promotes apoptosis in lung cancer cells by inhibiting the FA pathway (Wang et al., 2015; Fan et al., 2021). In addition, FANCD2 negatively regulates ferroptosis by regulating iron metabolism-related genes and/or protein expression and lipid peroxidation (Song et al., 2016). However, the molecular mechanisms of FANCD2 governing the regulation of the immune response in LUAD are still unclear.

This study aimed to explore the association between *FANCD2* expression and clinical information and prognosis in LUAD. The results show that FANCD2 expression regulates the level of tumor-infiltrating immune cells through multiple pathways, which contributes to the formation of the immunosuppressive microenvironment. Therefore, this study anticipates promising potential therapeutic strategies for LUAD based on FANCD2.

## Materials and Methods

### Identification of Ferroptosis- and NSCLC-Related Targets

Ferroptosis-related targets were identified from FerrDb (http://www.zhounan.org/ferrdb), which has data on 253 regulators, 111 markers, and 95 ferroptosis-associated diseases. NSCLC-related targets were identified from the GEO database, which contains many bioinformatics datasets from the National Center of Biotechnology Information (https://www.ncbi.nlm.nih.gov/geo/). Three gene expression datasets (GSE75037, GSE19188, and GSE116959) derived from human NSCLC tissues and adjacent normal tissues were included. Genes with an adjusted *p* < 0.01 and |log_2_(fold-change)|>2 were defined as differentially expressed genes (DEGs), which were considered to play essential roles in NSCLC progression and defined as key targets for inducing ferroptosis to treat NSCLC.

### Data Acquisition and Analysis

TCGA (https://portal.gdc.cancer.gov/) is a large-scale and open-access cancer genomic database. All transcriptome RNA-seq data (*n* = 1,145) and equivalent clinical data related to NSCLC were downloaded. Based on pathological characteristics, all patients were divided into LUAD (*n* = 516) and LUSC (*n* = 493) groups. Subgroup analysis was performed to investigate the effect of FANCD2 on the pathological stage and outcome. Three independent cohorts including tumor tissues and control samples of NSCLC patients from the GEO databases GSE75037 (*n* = 166), GSE19188 (*n* = 156), and GSE116959 (*n* = 68) were used to confirm the findings from TCGA datasets. HPA (https://www.proteinatlas.org/) is a comprehensive resource database of the human proteome, and the protein levels of FANCD2 in normal lung tissues and LUAD and LUSC tissues compared according to immunohistochemistry (IHC) results.

### Survival and Statistical Analyses

Based on the median expression of *FANCD2*, patients with LUAD and LUSC were divided into a high and a low group. To identify whether the FANCD2 expression level influenced LUAD and LUSC patient clinical survival, Kaplan–Meier (KM) survival curves were generated to estimate its prognostic significance.

### Univariate and Multivariate Logistic Regression Analyses

To further identify the prognostic value of FANCD2 in LUAD, Cox analyses were adopted to evaluate the relationships between various clinical characteristics and prognosis. Univariate Cox analysis was conducted for every variable comparing the expression level of FANCD2 and patient overall survival (OS) in each cohort to confirm their association with LUAD prognosis. Subsequently, multivariate Cox analysis, including all variables, was used to evaluate whether FANCD2 was an independent prognostic factor for LUAD patient outcome.

### Genetic Alteration and Interaction Network Analyses

Based on cBioPortal (http://cbioportal.org), an open-access multidimensional cancer genomics resource, FANCD2 alterations were analyzed in LUAD samples collected from TCGA. GeneMANIA (http://genemania.org/) and STRING (https://string-preview.org/) are source websites used to construct gene–gene and protein–protein interaction networks, respectively. Both were applied to investigate FANCD2-related genes and proteins.

### Single-Cell Analysis

The CancerSEA (http://biocc.hrbmu.edu.cn/CancerSEA/home.jsp) database provides a cancer single-cell functional state atlas. This study used CancerSEA to explore the function of FANCD2-regulated genes, as well as the correlation between FANCD2 expression levels and these functions.

### TISIDB Database Analysis

The TISIDB (http://cis.hku.hk/TISIDB/) web portal was used to probe tumor-immune interactions, which was applied to evaluate correlations between FANCD2 and immune-suppressive genes and immune-activating genes in this study.

### TIMER Database Analysis

TIMER (https://cistrome.shinyapps.io/timer/) is a web server for the systematic analysis of six tumor-infiltrating immune subsets across diverse cancer types. Here, the correlation between FANCD2 expression and the infiltration of B cells, CD8^+^ T cells, CD4^+^ T cells, dendritic cells (DCs), macrophages, and neutrophils was assessed in Patients with LUAD. Additionally, the association between FANCD2 expression and tumor purity was tested, according to the “Correlation” module of TIMER and the tumor purity-corrected partial.

### ssGSEA and CIBERSORT Analysis

In total, 24 types of FANCD2-related immune cells were acquired from ssGSEA using the GSVA package in R (4.0.3). Pearson correction analysis was performed to further assess the relevance and enrichment scores between FANCD2 and the different immune cells. CIBERSORT (http://cibersort.stanford.edu/), a deconvolution algorithm based on gene expression, was used to reveal the relevance of 24 types of immune cells in LUAD.

### GEPIA Database Analysis

GEPIA (http://gepia.cancer-pku.cn/index.html) covers thousands of tumors and normal samples from TCGA and the Genotype-Tissue Expression Project (GTEx, http://www.gtexportal.org/home/index.html). It focuses on the analysis of RNA-seq data. The association between FANCD2 levels and multiple markers for various immune cells was evaluated according to the GEPIA database.

### Statistical Analysis

Student’s *t*-tests and Wilcoxon tests were performed for assessing differences between two groups, and a Kruskal-Wallis test was used for more than two groups. To evaluate patient survival, KM curves, as well as univariate and multivariate logistic regression analyses, were conducted. Spearmen or Pearson correlations were applied to calculate the relationship between FANCD2 and immune infiltration. Data analyses were based on R (v4.0.3), and *p* < 0.05 was considered to indicate statistical significance.

## Results

### Hub Genes Associated With Ferroptosis and NSCLC

**FIGURE 1:**
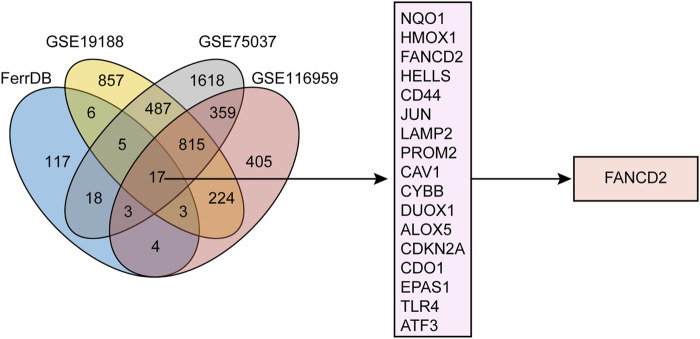
Workflow of target screening. Venn diagram showing differentially expressed genes (DEGs) in NSCLC from three GEO cohorts alongside ferroptosis-related genes.

In total, 173 genes in the FerrDb and 2,414, 3,322, and 1,830 DEGs in GSE19188, GSE75037, and GSE116959, respectively, were identified. As shown in Figure 1, 17 candidate genes comprised the intersection of the four datasets. Of these, the top five targets were nicotinamide adenine dinucleotide phosphate (NAD[P]H) dehydrogenase (quinone 1) (*NQO1*), heme oxygenase 1 (*HMOX1*), *FANCD2*, *helicase*, lymphoid specific (*HELLS*), and cluster of differentiation 44 (*CD44*)*.* Notably, in addition to participating in ferroptosis, FANCD2 is involved in repairing DNA lesions and ensuring accurate DNA replication (Wang et al., 2015; Yang et al., 2016; Li et al., 2020). Owing to numerous pathways that FANCD2 could regulate in the occurrence and development of cancer, it deserves deeper investigation. Therefore, *FANCD2* was chosen for further analysis.

### Patient Characteristics

The RNA-seq expression data and clinical prognostic information of 535 LUAD patients and 493 LUSC patients with were obtained from the TCGA database. The clinical information of patients with LUAD or LUSC with high or low FANCD2 expression levels is summarized in Table 1, including age, sex, smoking status, pathologic stage (T, N, or M), histologic grade, OS, disease-specific survival (DSS), and the progression-free interval (PFI).

**TABLE 1:** Clinical characteristics of lung adenocarcinoma (LUAD) and lung squamous carcinoma (LUSC) patients with FANCD2 high or low expression based on TCGA database.

Clinical factors		LUAD			LUSC		
		Low	High	*p* Value	Low	High	*p* Value
Age, n (%)	Median (IQR)	67 (59, 74)	64 (59, 72)	0.036	69 (63, 74)	67 (60, 73)	0.033
	≤65	115 (22.3%)	140 (27.1%)	0.043	87 (17.6%)	104 (21.1%)	0.170
	>65	142 (27.5%)	119 (23.1%)		158 (32%)	144 (29.2%)	
Gender, n (%)	Female	158 (29.5%)	128 (23.9%)		79 (15.7%)	52 (10.4%)	0.008
	Male	109 (20.4%)	140 (26.2%)		172 (34.3%)	199 (39.6%)	
Smoker, n (%)	No	47 (9%)	28 (5.4%)	0.018	8 (1.6%)	10 (2%)	0.797
	Yes	210 (40.3%)	236 (45.3%)		238 (48.6%)	234 (47.8%)	
T stage, n (%)	T1	98 (18.4%)	77 (14.5%)	0.031	61 (12.2%)	53 (10.6%)	0.416
	T2	127 (23.9%)	162 (30.5%)		138 (27.5%)	156 (31.1%)	
	T3	29 (5.5%)	20 (3.8%)		40 (8%)	31 (6.2%)	
	T4	11 (2.1%)	8 (1.5%)		12 (2.4%)	11 (2.2%)	
N stage, n (%)	N0	175 (33.7%)	173 (33.3%)	0.580	167 (33.7%)	153 (30.8%)	0.321
	N1	44 (8.5%)	51 (9.8%)		58 (11.7%)	73 (14.7%)	
	N2	38 (7.3%)	36 (6.9%)		17 (3.4%)	23 (4.6%)	
	N3	0 (0%)	2 (0.4%)		3 (0.6%)	2 (0.4%)	
M stage, n (%)	M0	187 (48.4%)	174 (45.1%)	0.088	197 (47%)	215 (51.3%)	0.270
	M1	8 (2.1%)	17 (4.4%)		5 (1.2%)	2 (0.5%)	
Pathologic stage, n (%)	Stage I	147 (27.9%)	147 (27.9%)	0.455	128 (25.7%)	117 (23.5%)	0.491
	Stage II	61 (11.6%)	62 (11.8%)		76 (15.3%)	86 (17.3%)	
	Stage III	44 (8.3%)	40 (7.6%)		41 (8.2%)	43 (8.6%)	
	Stage IV	9 (1.7%)	17 (3.2%)		5 (1%)	2 (0.4%)	
OS event, n (%)	Alive	180 (33.6%)	163 (30.5%)	0.134	144 (28.7%)	142 (28.3%)	0.928
	Dead	87 (16.3%)	105 (19.6%)		107 (21.3%)	109 (21.7%)	0.777
DSS event, n (%)	Alive	198 (39.7%)	181 (36.3%)	0.079	183 (40.7%)	178 (39.6%)	
	Dead	51 (10.2%)	69 (13.8%)		43 (9.6%)	46 (10.2%)	
PFI event, n (%)	Alive	162 (30.3%)	147 (27.5%)		177 (35.3%)	177 (35.3%)	1.000
	Dead	105 (19.6%)	121 (22.6%)		74 (14.7%)	74 (14.7%)	

### FANCD2 Expression Is Higher in Tumor Samples Than in Normal Tissues

**FIGURE 2:**
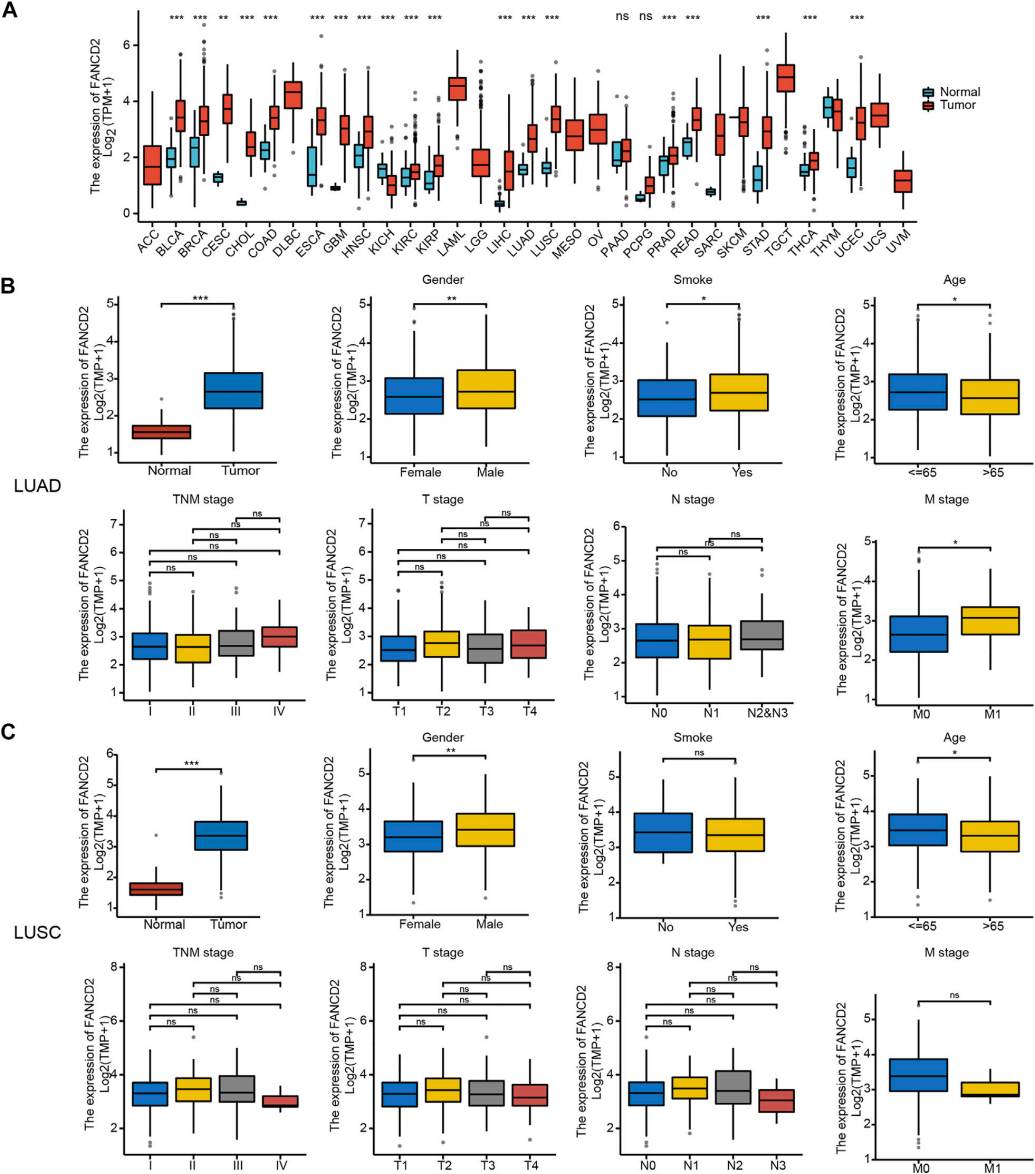
Pan-cancer FANCD2 expression status and clinical characteristics in the LUAD and LUSC sub-groups. **(A)** FANCD2 expression status in different tumor tissues and adjacent normal tissues. **(B,C)** Comparison of FANCD2 expression levels with different clinical characteristics. *p < 0.05; **p < 0.01; ***p < 0.001.

According to the TCGA and GTEx databases, *FANCD2* exhibited higher expression in 19 types of tumor tissues, including LUAD and LUSC, than in adjacent normal samples in the TCGA database (Figure 2A). Correlation analysis showed that *FANCD2* mRNA expression was significantly higher in older patients (age>65 years, *p* < 0.05), in males (*p* < 0.01), and in smoking patients (*p* < 0.05), as well as in patients with higher M stage (*p* < 0.05, Figure 2B). Meanwhile, *FANCD2* expression was not related to pathological stage, T stage, or N stage (Figure 2C). *FANCD2* expression exhibited similar tendencies concerning the age and gender of patients with LUSC, but there were no obvious differences between *FANCD2* expression and other variables.

**FIGURE 3:**
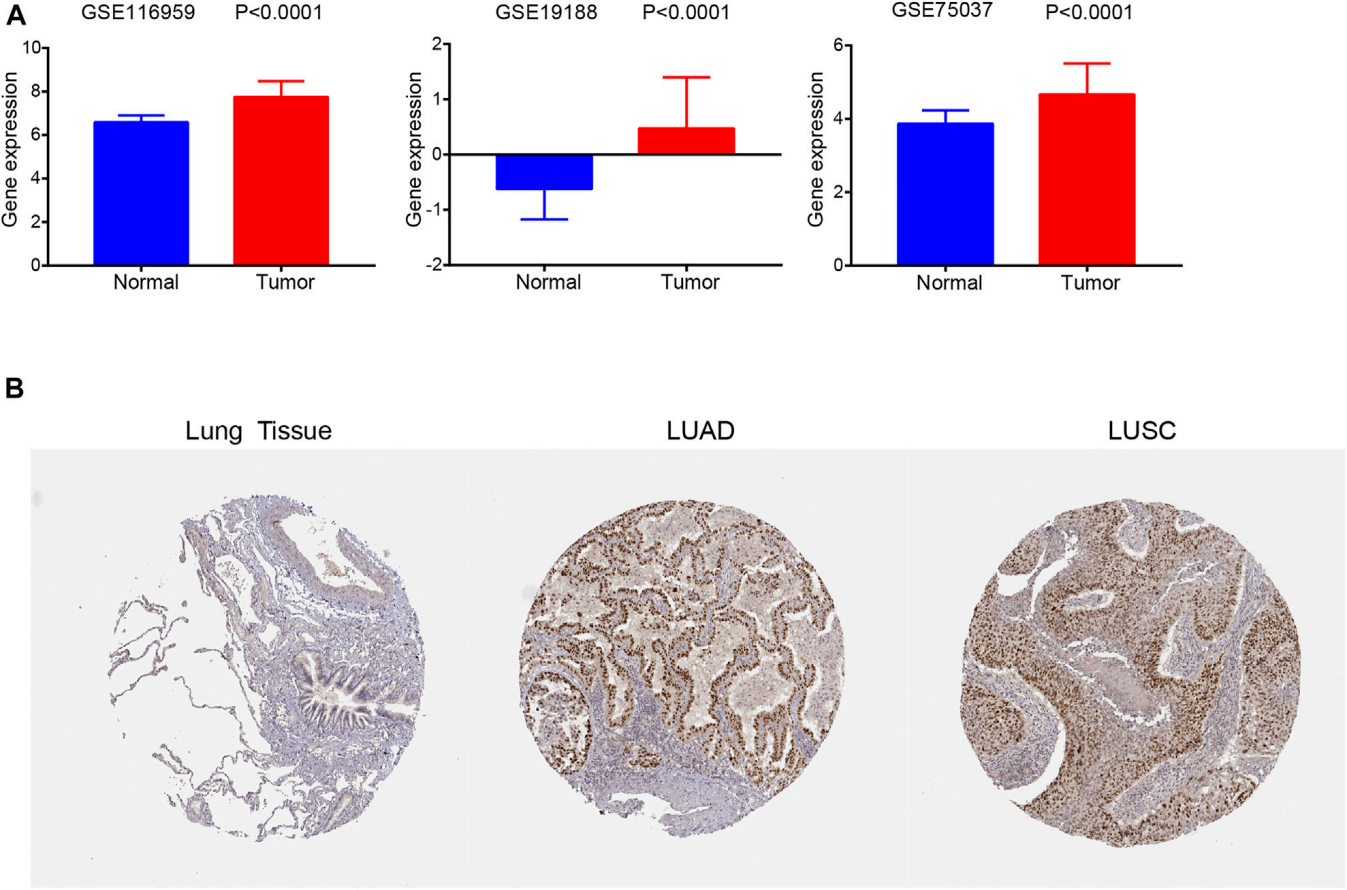
FANCD2 expression in GEO datasets and the HPA database. **(A)** *FANCD2* mRNA expression levels were higher in tumors than in normal tissues based on GSE19188 and GSE116959 datasets; *p* < 0.01. **(B)** FANCD2 protein levels in LUAD and LUSC tissues and normal samples based on the HPA database.

Similarly, *FANCD2* was also significantly elevated in NSCLC tissues compared to levels in normal tissues based on GSE19188, GSE75037, and GSE116959 datasets (all *p* < 0.001, Figure 3A). In addition, the IHC results revealed that FANCD2 was overexpressed in LUSC and LUAD tissue in comparison with that in normal tissue according to HPA databases (Figure 3B). These results indicated the gene and protein expression levels of FANCD2 are significantly higher in LUAD and LUSC tissues.

### High FANCD2 mRNA Expression Is Related to Short OS in Patients With LUAD

**FIGURE 4:**
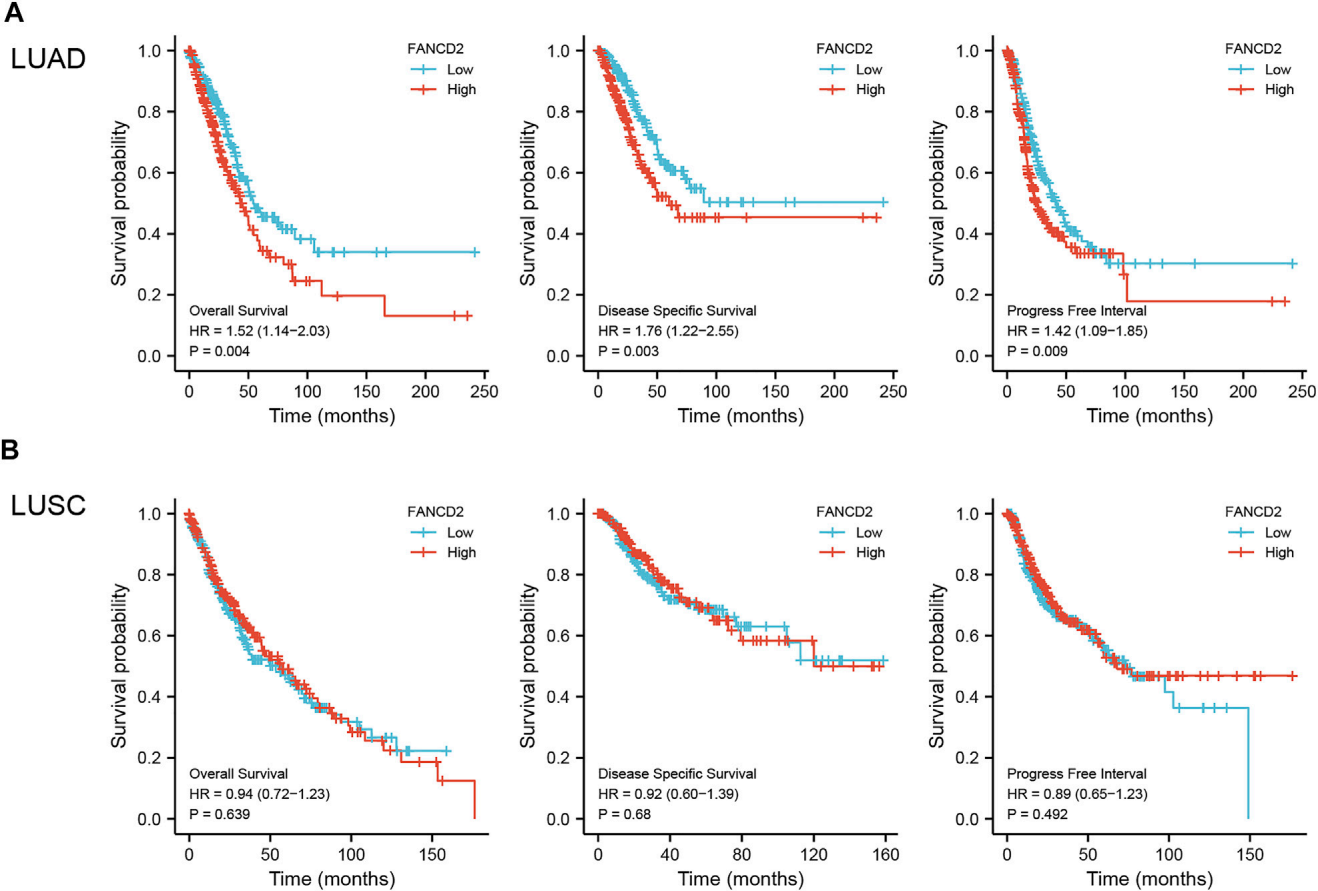
Kaplan–Meier (K-M) survival curves showing the association between FANCD2 expression levels and overall survival (OS), disease-specific survival (DSS), and the progression-free interval (PFI) for LUAD **(A)** and LUSC **(B)** patients; *p* < 0.05 was used to assess differences.

**FIGURE 5:**
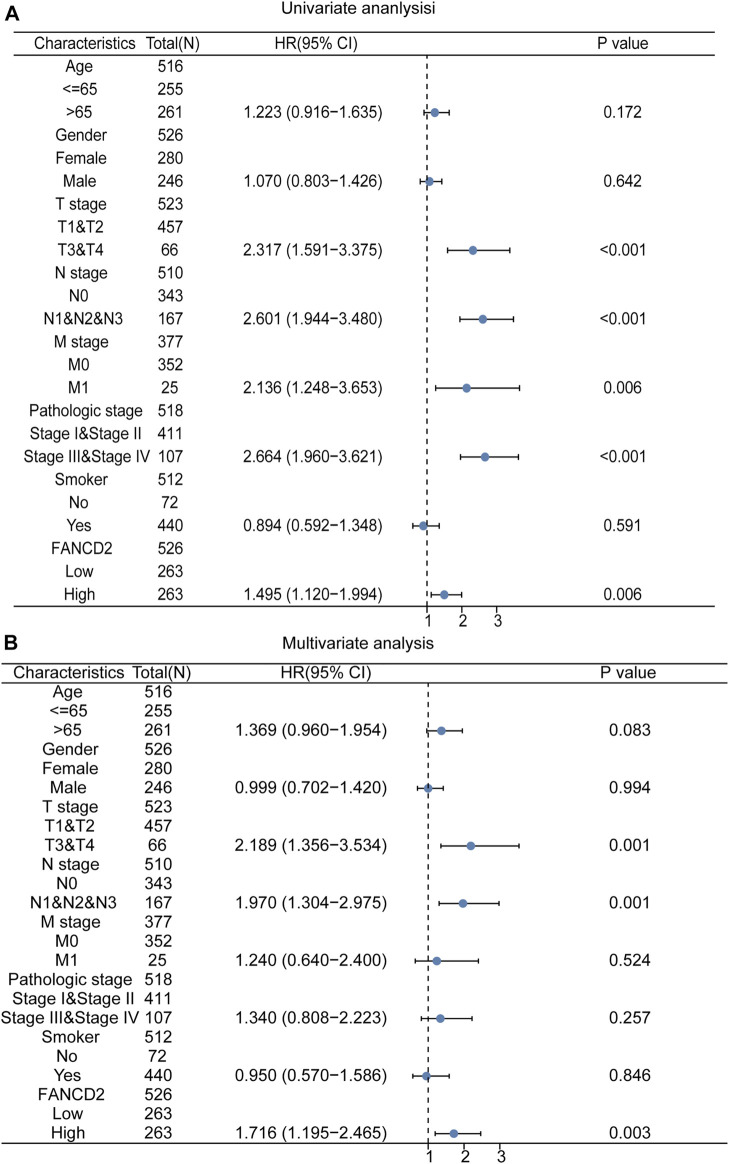
Univariate **(A)** and multivariate **(B)** analyses of FANCD2 expression and important clinicopathological parameters concerning prognosis among Patients with LUAD.

As shown in Figure 4A, the 20-year OS, DSS, and PFI rates of Patients with LUAD were remarkably higher with low *FANCD2* expression compared to those with high *FANCD2* expression (*p* = 0.04, 0.03, and 0.09 respectively). However, there was no significant difference between *FANCD2* expression and OS (*p* = 0.639), DSS (*p* = 0.68), and PFI (*p* = 0.492) in LUSC patients (Figure 4B). Univariate analysis showed that both high *FANCD2* expression and high pathological grade and stage (TNM) were related to poor OS in Patients with LUAD (Figure 5A). The results of multivariate Cox regression analysis suggested that high *FANCD2* expression was an independent predictor of OS (HR = 1.716, 95% CI = 1.195–2.465, *p* < 0.01, Figure 5B). Therefore, *FANCD2* was considered a risk factor in predicting a worse prognosis.

### Genetic Alteration and Interaction Network Analyses of FANCD2

**FIGURE 6:**
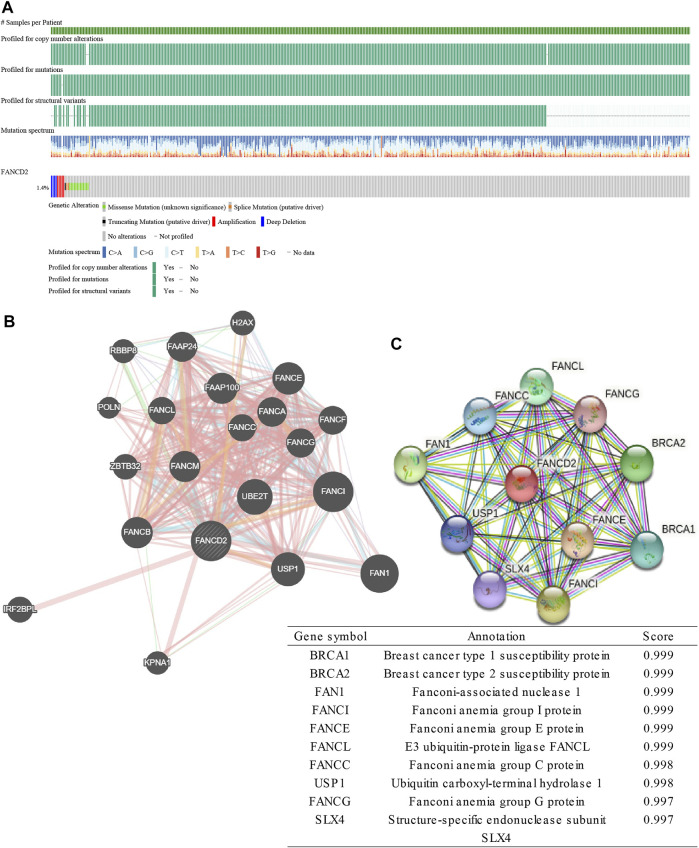
Genomic alterations of FANCD2 and the interaction network and protein interaction network of FANCD2. **(A)** Genomic alterations of FANCD2 based on cBioPortal. **(B)** The gene–gene interaction network of FANCD2, as constructed with GeneMANIA. **(C)** The protein-protein network of FANCD2 is derived from STRING.

The cBioportal online tool was then performed to explore the types and frequencies of *FANCD2* alterations in the patients with LUAD. Results revealed that *FANCD2* was highly conserved (only a 1.4% frequency of genomic alterations; Figure 6A). Subsequently, the interaction networks showed 20 genes (Figure 6B) and 10 proteins (Figure 6C) with the highest relevance to FANCD2. FANCI, FANCL, FAN1, FANCE, FANCC, and USP1 appeared in two networks, for which correlation scores were 0.999, 0.999, 0.999, 0.999, 0.998, and 0.998, respectively, in PPI network (Figure 6C). These genes/proteins belong to the FA family. FANCD2 and FANCI, via their heterodimer, serve a function in the FA pathway, in which the dimer, monoubiquitylated by the FA core complex and an E2-E3 ubiquitin ligase, participates in the recruitment of DNA repair effectors to chromatin lesions to resolve DNA damage (Nalepa and Clapp, 2018). In this process, FANCE and FANCC form part of the FA core complex; FANCE encodes an E3 ubiquitin ligase that monoubiquitylates FANCD2 and FANCI; USP1 and UAF1 regulate the deubiquitination of the FANCD2-FANCI heterodimer (Liu et al., 2010; Nalepa and Clapp, 2018; Lemonidis et al., 2021).

### Functions of FANCD2 in LUAD

**FIGURE 7:**
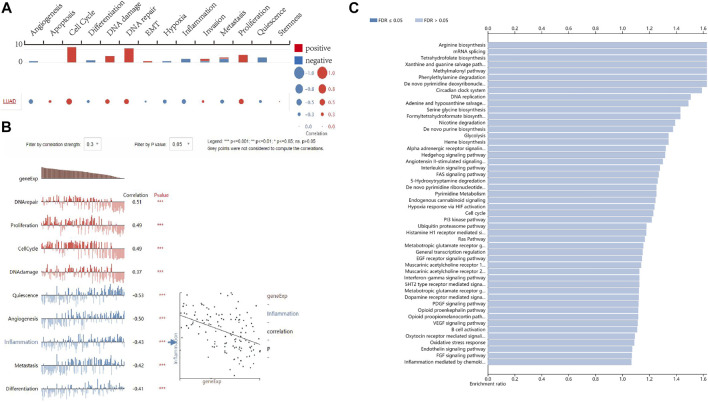
The function of FANCD2 in LUAD. **(A,B)** The single-cell analysis shows that FANCD2 is involved in the cell cycle, DNA repair, proliferation, angiogenesis, quiescence, inflammation, metastasis, and differentiation. **(C)** The biological processes related to FANCD2 are based on ORA.

To better understand the relevance of FANCD2 expression in LUAD and potential mechanism, single-cell analysis was utilized to explore the associated functional states based on the CancerSEA database. The results suggested that FANCD2 expression was correlated with 14 functional states, including angiogenesis, apoptosis, cell cycle, differentiation, DNA damage, DNA repair, EMT, hypoxia, inflammation, invasion, metastasis, proliferation, quiescence, and stemness (Figure 7A). In addition, FANCD2 was found to be mainly positively associated with cell cycle, DNA repair, DNA damage, and proliferation but negatively correlated with angiogenesis, quiescence, inflammation, metastasis, and differentiation (all *p* < 0.001, Figure 7B). Moreover, over-representation analysis (ORA) illustrated that FANCD2 participated in the interleukin signaling pathway, *de novo* pyrimidine deoxyribonucleotide biosynthesis, DNA replication, *de novo* purine biosynthesis, arginine biosynthesis, angiotensin II-stimulated signaling through G proteins and beta-arrestin, the circadian clock system, and the EGF receptor signaling pathway (all *p* < 0.05, Figure 7C). Notably, the interleukin signaling pathway had the highest correlation with FANCD2. It is well-known that interleukin family members play important roles in the immune response and inflammation. These results indicate that FANCD2 might participate in immune response and inflammation *via* the interleukin signaling pathway.

### Association Between FANCD2 Expression and Immune-Inhibitory and Immune-Stimulatory Functions

**FIGURE 8:**
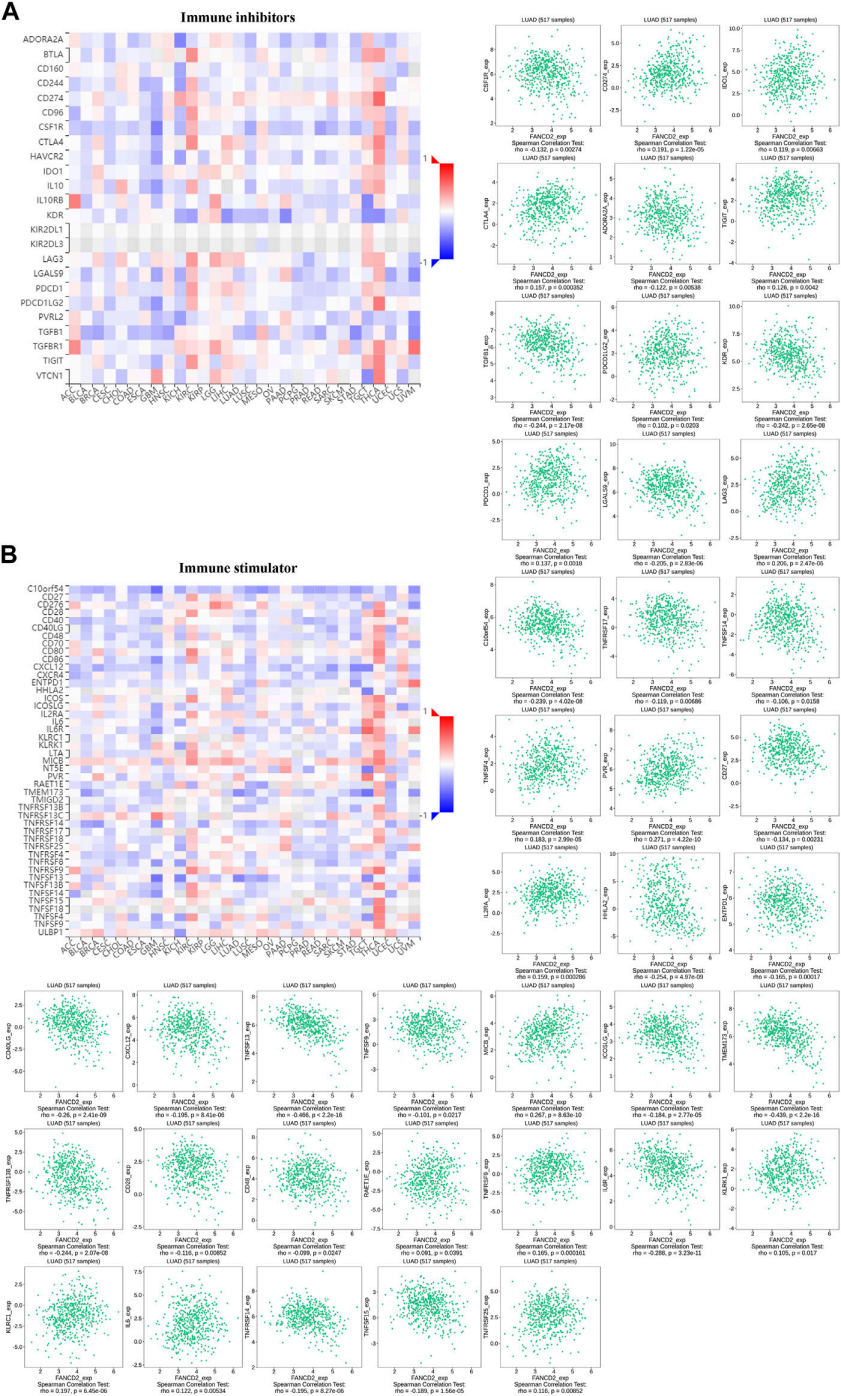
Co-expression analysis of FANCD2 with immunoinhibitory **(A)** and immunostimulatory genes **(B)** in the pan-cancer database, as well as detailed information on LUAD.

A co-expression study was performed to investigate the relationship between FANCD2 expression and immunomodulators based on TISIDB, including immune inhibitors and immunostimulators. Heatmaps illustrated the association between FANCD2 expression and 12 immunoinhibitors and 28 immunostimulators across 30 tumors (Figure 8). Among these immunoinhibitors, FANCD2 expression had positive associations with CD274 (Cor = 0.191, *p* = 1.22e-05), CTLA4 (Cor = 0.157, *p* = 3.52e-04), LAG3 (Cor=(Cor = 0.206, *p* = 2.47e-06), and PDCD1 (Cor = 0.137, *p* = 1.8e-03) in LUAD (Figure 8A). Regarding various immunostimulators, FANCD2 had negative associations with TNFSF13 (Cor = −0.466, *p* < 2.2e-16), TMEM173 (Cor = −0.439, *p* < 2.2e-16), CD40LG (Cor = −0.26, *p* = 2.41e-09), HHLA2 (Cor = −0.254, *p* = 4.97e-09), and IL6R (Cor = −0.288, *p* = 3.23e-11) (Figure 8B). The above results suggest that FANCD2 is involved in regulating these immunomodulators.

### Correlation Between FANCD2 Expression and Infiltrating Immune Cells

**FIGURE 9:**
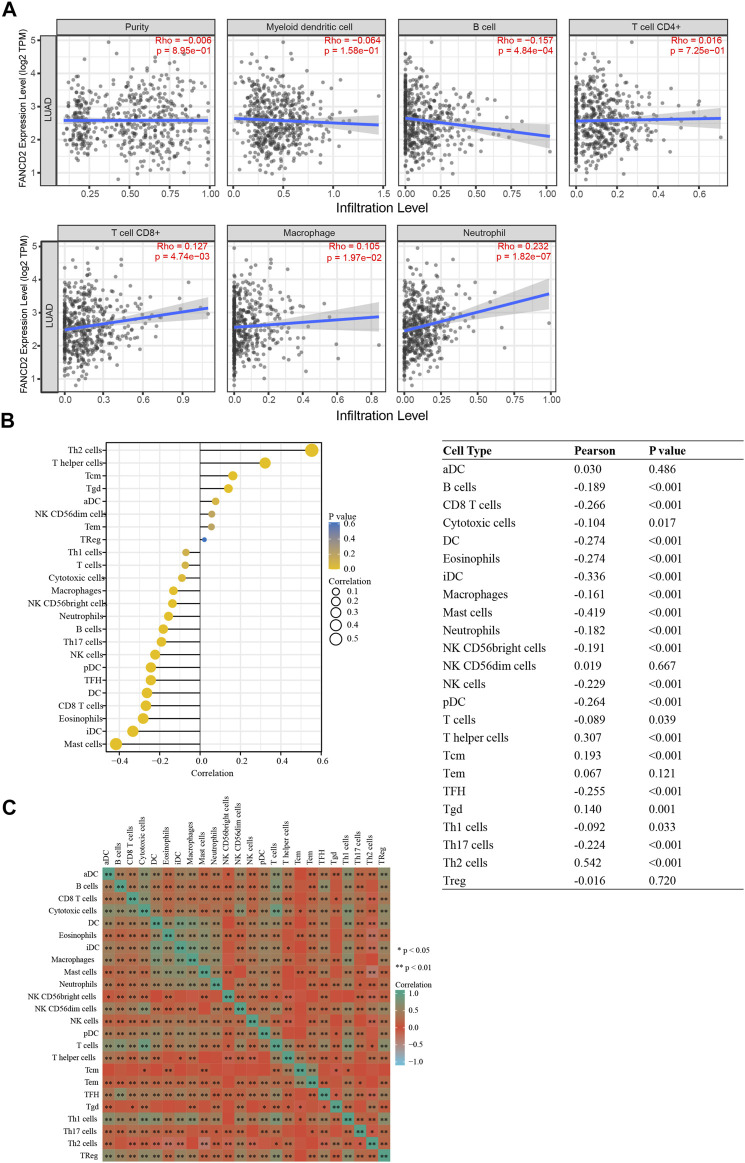
Correlation between FANCD2 expression and immune infiltration levels in LUAD. **(A)** Correlation analysis of FANCD2 expression and the infiltration of six types of immune cells based on TIMER. **(B)** Forest plots show that FANCD2 expression was positively correlated with 8 types of immune cells and negatively correlated with 16 subsets of immune cells. The sizes of dots represent the absolute value of Pearson r. **(C)** Heatmap showing the relationship among 24 types of immune cells in LUAD. *p < 0.05, **p < 0.01.

Tumor-infiltrating lymphocytes are associated with the prognosis of patients with multiple cancers (Salgado and Loi, 2018). As shown in Figure 9A, FANCD2 expression levels were strongly associated with levels of infiltrating B cells (Cor = −0.157, *p* = 4.84e-04), CD8^+^ T cells (Cor = 0.127, *p* = 4.74e-03), macrophages (Cor = 0.105, *p* = 1.97e-02), and neutrophils (Cor = 0.232, *p* = 1.82e-07) in LUAD cases. However, there was no association between FANCD2 expression and tumor purity, DCs, or CD4^+^ T cells. To gain more insight into the relationship between FANCD2 expression and immune infiltration, this study assessed subjects based on 24 types of infiltrating immune cells using the ssGSEA database (Figure 9B). Specifically, FANCD2 was negatively related to B cells, CD8^+^ T cells, DC cells, macrophages, eosinophils, Idc (interdendritic) cells, mast cells, neutrophils, NK CD56 bright cells, pDC (plasmacytoid dendritic) cells, TFH cells, TH17 cells, and NK cells but was positively related to Th2 cells, T helper cells, and Tcm cells (all *p* < 0.001). Moreover, the heat map showed that most subpopulations among the 24 types of immune cells had moderate to strong relationships (Figure 9C). These findings evealed that FANCD2 plays an important role in immune infiltration in LUAD.

GEPIA and TIMER databases were used to further assess the correlation between FANCD2 and the marker sets of diverse immune cells in LUAD. Table 2 shows that multiple markers of immune cells were significantly related to FANCD2 expression, including Th2 (GATA3), Th9 (TGFBR2), Th17 (IL-21R), Treg (FOXP3, CD25, CCR8), T cell exhaustion (PD-1, CTLA4, LAG3), tumor-associated macrophages (TAMs) (CD80, CCR5), and DCs (CD1C, CD141). These results implied that FANCD2 might affect the function of immune cells by modulating marker gene expression.

**TABLE 2:** Correlation between FANCD2 levels and markers of immune cells based on TIMER and GEPIA databases.

Cell type	Gene marker	None		Purity		Tumor		Normal	
		Cor	P	Cor	P	R	P	R	P
B cell	CD19	−0.003	0.9410	0.004	0.9210	−0.085	0.063	0.41	0.0011^**^
	CD20(KRT20)	0.027	0.5440	0.021	0.6440	0.071	0.12	−0.039	0.77
	CD38	0.049	0.2720	0.064	0.1560	−0.0043	0.92	0.14	0.29
CD8^+^ T cell	CD8A	0.201	^***^	0.231	^***^	0.078	0.085	0.11	0.41
	CD8B	0.192	^***^	0.202	^***^	0.12	0.0082^**^	0.085	0.52
	BCL6	0.019	0.6600	0.024	0.5960	0.056	0.22	0.13	0.31
	ICOS	0.162	0.0002^***^	0.202	^***^	0.069	0.13	0.32	0.014^**^
	CXCR5	−0.013	0.7760	−0.005	0.9150	−0.098	0.032	0.4	0.0017^**^
Th1	T-bet (TBX21)	0.156	0.0004^***^	0.184	^***^	0.035	0.44	−0.033	0.8
	STAT4	0.081	0.0646	0.098	0.0291	0.033	0.47	0.31	0.18
	IL12RB2	0.457	^***^	0.483	^***^	0.22	^***^	0.14	0.29
	WSX1(IL27RA)	−0.047	0.2900	−0.043	0.3410	−0.058	0.21	0.24	0.071
	STAT1	0.44	^***^	0.472	^***^	0.3	^***^	0.12	0.36
	IFN-γ(IFNG)	0.311	^***^	0.337	^***^	0.19	0.000026^***^	0.075	0.57
	TNF-α(TNF)	0.103	0.0194^*^	0.124	0.0056^**^	0.055	0.23	0.33	0.011^*^
Th2	GATA3	0.174	0.0001^***^	0.204	^***^	0.19	0.000026^***^	0.03	0.82
	CCR3	−0.02	0.6430	0.001	0.9890	−0.015	0.74	0.11	0.41
	STAT6	−0.07	0.1120	−0.077	0.0858	−0.028	0.55	0.27	0.042^*^
	STAT5A	0.055	0.2140	0.076	0.0925	−0.0088	0.85	0.44	0.00056^**^
Th9	TGFBR2	−0.13	0.0032^**^	−0.123	0.0064^***^	−0.12	0.0077^***^	−0.19	0.14
	IRF4	0.062	0.1620	0.077	0.0858	−0.068	0.13	0.44	0.00053^**^
	PU.1(SPI1)	−0.039	0.3770	−0.038	0.4040	−0.11	0.015^*^	0.24	0.071
Th17	STAT3	0.023	0.5980	0.021	0.6350	0.021	0.64	0.12	0.38
	IL-21R	0.149	0.0007^***^	0.188	^***^	0.037	0.41	0.26	0.05
	IL-23R	0.002	0.9580	0.006	0.9010	−0.033	0.47	0.11	0.39
	IL-17A	0.089	0.0436^*^	0.089	0.0494	0.073	0.11	0.17	0.19
Th22	CCR10	0.094	0.0321^*^	0.08	0.0774	0.11	0.013^*^	−0.086	0.52
	AHR	−0.014	0.7550	−0.014	0.7500	−0.012	0.79	0.003	0.98
Treg	FOXP3	0.157	0.0004^***^	0.183	***	0.04	0.38	0.42	0.001^**^
	CD25(IL2RA)	0.24	^***^	0.271	***	0.15	0.00068	0.11	0.39
	CCR8	0.183	^***^	0.214	***	0.064	0.16	0.21	0.11
T cell exhaustion	PD-1(PDCD1)	0.219	^***^	0.259	***	0.083	0.068	0.38	0.0029^**^
	CTLA4	0.24	^***^	0.29	***	0.14	0.0029	0.27	0.04
	LAG3	0.265	^***^	0.286	***	0.12	0.0086	0.43	0.00071^***^
	TIM-3(HAVCR2)	0.074	0.0951	0.088	0.0516	−0.033	0.46	0.097	0.46
Macrophage	CD68	0.034	0.4350	0.044	0.3340	−0.032	0.49	0.15	0.26
	CD11b (ITGAM)	0.006	0.9000	0.019	0.6670	−0.026	0.57	0.23	0.074
M1	INOS(NOS2)	0.042	0.3420	0.029	0.5260	0.048	0.29	−0.25	0.058
	IRF5	0.125	0.0045^**^	0.134	0.0028	0.087	0.057	0.4	0.0016^**^
	COX2(PTGS2)	0.016	0.7150	0	0.9920	0.02	0.66	−0.048	0.72
M2	CD16	0.042	0.3440	0.055	0.2230	−0.0086	0.85	0.065	0.62
	ARG1	−0.001	0.9830	−0.011	0.8150	0.059	0.19	−0.038	0.77
	MRC1	−0.083	0.0588	−0.079	0.0786	−0.075	0.1	0.38	0.0031^**^
	MS4A4A	−0.001	0.9860	0.007	0.8760	−0.083	0.069	0.25	0.06
TAM	CCL2	0.074	0.0921	0.081	0.0739	0.0014	0.97	−0.015	0.91
	CD80	0.129	0.0033^**^	0.153	0.0006	0.079	0.082	0.15	0.25
	CD86	0.078	0.0755	0.095	0.0355	−0.028	0.54	0.22	0.1
	CCR5	0.14	0.0014	0.177	0.0001	0.056	0.22	0.27	0.04^*^
Monocyte	CD14	0.041	0.3530	0.058	0.1960	−0.06	0.19	−0.12	0.35
	CD16(FCGR3B)	0.042	0.3440	0.055	0.2230	−.0086	0.85	0.065	0.62
	CD115(CSF1R)	0.011	0.8050	0.026	0.5690	−0.047	0.3	0.34	0.00088^**^
Neutrophil	CD66b (CEACAM8)	−0.23	^***^	−0.229	***	−0.1	0.025	−0.013	0.92
	CD15(FUT4)	0.058	0.1890	0.047	0.2940	0.15	0.0013	0.15	0.26
	CD11b (ITGAM)	0.006	0.9000	0.019	0.6670	−0.026	0.57	0.23	0.074
Natural killer cell	XCL1	0.225	^***^	0.261	***	0.2	0.0000058	−0.17	0.19
	CD7	0.191	^***^	0.219	***	0.063	0.17	0.16	0.22
	KIR3DL1	0.035	0.4270	0.042	0.3560	0.00052	0.99	−0.19	0.16
Dendritic cell	CD1C(BDCA-1)	−0.339	^***^	−0.344	***	−0.23	0.00000028	0.11	0.43
	CD141 (THBD)	−0.183	^***^	−0.188	***	−0.075	0.1	−0.12	0.38
	CD11c	0.133	0.0026^**^	0.154	0.0006	0.011	0.81	0.35	0.0071^**^

correlation without adjustment, Purity: correlation adjusted by purity, Cor, correlation coefficient. *p < 0.05, **p < 0.01, and ***p < 0.001.

## Discussion

LUAD is the most common subtype of lung cancer. Currently, the efficacy of surgery, radiotherapy, chemotherapy, and targeted therapy is not satisfying. Notably, ferroptosis is a novel form of cell death, and an increasing body of research has confirmed that it plays a crucial role in anti-tumor treatment, especially in LUAD (Ma et al., 2021; Zhang X. et al., 2021). Moreover, a connection between ferroptosis and cell immunity and cancer immunotherapy has been shown, but the underlying mechanism is not clear (Wang et al., 2019). The latest study showed that 76.9% of ferroptosis-related genes are differentially expressed between LUAD tumor tissues and adjacent normal tissues, and some of these DEGs were determined to be remarkably associated with OS (Gao et al., 2021). Thus, ferroptosis-related genes are valuable prognostic markers for LUAD.

FANCD2, a member of the FA protein family, participates in the maintenance of genomic stability *via* the FA pathway. The interaction network derived from GeneMANIA and STRING shows a close connection between FANCD2 and other FA family genes/proteins. Until now, at least twenty-two FA proteins that form part of the FA core complex have been identified. The FA core complex participates in the recruitment and monoubiquitination of the heterodimer FANCD2-FANCI (Tsui and Crismani, 2019). Ubiquitylation of the FANCD2-FANCI heterodimer enables the recruitment of DNA repair effectors (Nalepa and Clapp, 2018), which participate in three classic DNA repair pathways, including nucleotide excision repair, homologous recombination, and mutagenic translesion synthesis (Moldovan and D'Andrea, 2009). As a nuclear protein, FANCD2 supports the maintenance of a stable genome, but it also has a negative regulatory role in ferroptosis, which is mainly involved in two biological pathways: iron accumulation and lipid peroxidation. Tumor cells with low FANCD2 expression undergo ferroptosis easily. Specifically, FANCD2 deficiency contributes to lipid peroxidation through a decrease in glutathione peroxidase 4 (GPX4), as well as the accumulation of iron through an increase in the expression of transferrin (TF), and a decrease in ferritin heavy chain 1 (FTH1) and SLC40A1 (Song et al., 2016).

**FIGURE 10:**
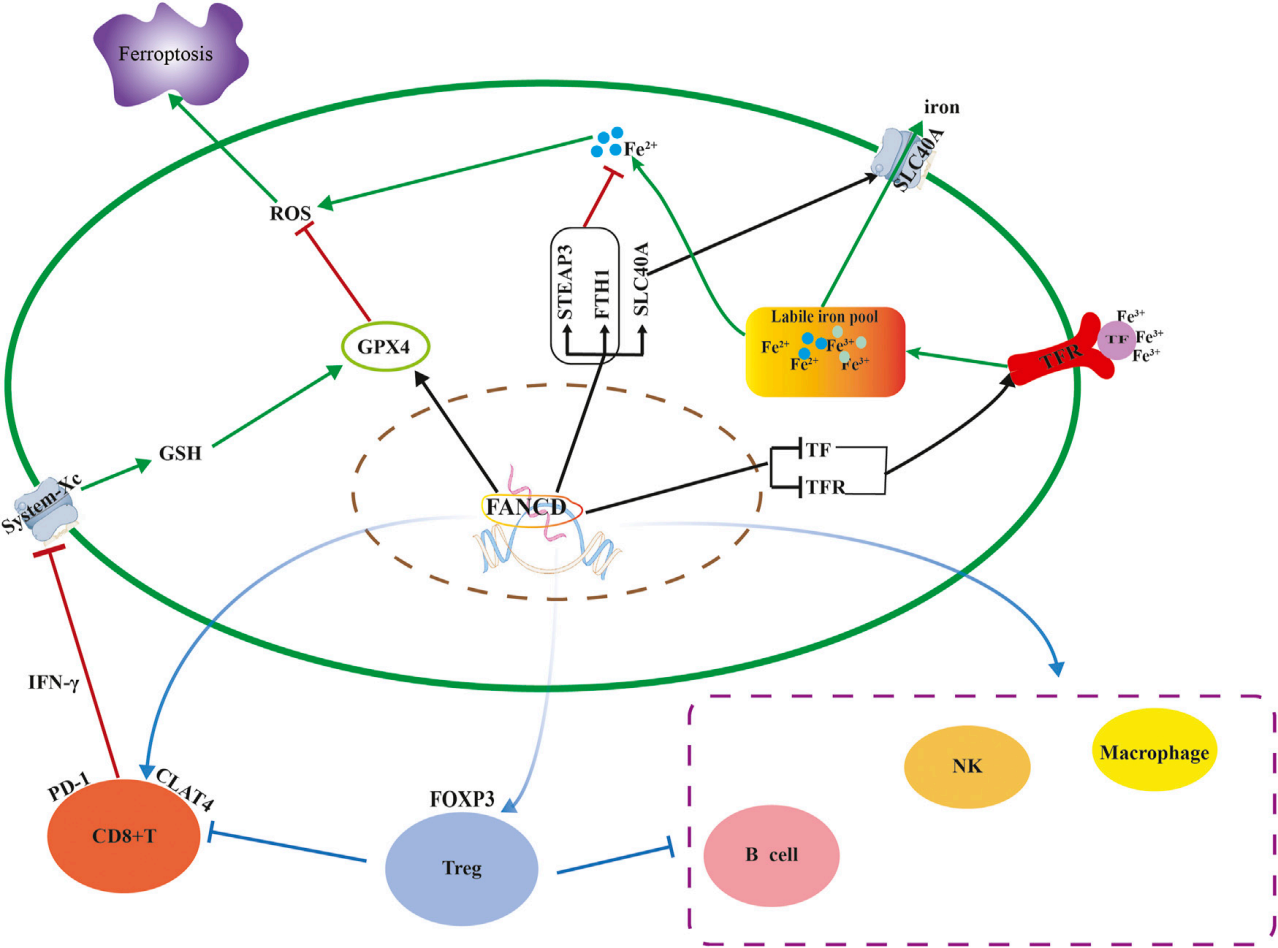
The mechanism of FANCD2 regulating ferroptosis and immune cell infiltration.

This study investigated the role of FANCD2 in LUAD progression and prognosis, as well as its relationship with immune cell infiltration (Figure 10). We observed that the mRNA and protein expression of FANCD2 were upregulated in LUAD samples compared to levels in normal tissues in the TCGA, GEO, and HPA databases, and patients with higher FANCD2 had shorter OS, poor DSS, and worse PFI based on KM plots in LUAD. Moreover, univariate and multivariate Cox analysis further confirmed that high expression of FANCD2 was an independent adverse prognostic factor, which was consistent with the results of Lei et al. (2020). Some research shows that inhibiting FANCD2 function or decreasing the expression of FANCD2 enhances the sensitivity of patients to chemotherapy, such as cisplatin, and the efficacy of treatments such as ICL agent and ionizing radiation (IR) (Wang et al., 2015; Yang et al., 2016). In addition, downregulated FANCD2 significantly inhibits tumor growth in nude mice (Fan et al., 2021). Therefore, high expression of FANCD2 was considered a poor prognostic biomarker for Patients with LUAD.

To be better able to elaborate on the molecular mechanisms of the highly conserved gene FANCD2 in LUAD, CancerSEA, and ORA were conducted to further investigate its function. CancerSEA at a single-cell level illustrated that FANCD2 participates in inflammation, and its intensity decreases with the expression of FANCD2; ORA results showed that FANCD2 participates in the interleukin signaling pathway. Numerous studies have confirmed that factors of the interleukin family, such as IL8, IL10, and IL17, are involved in immune responses and are associated with the outcome of patients (Schalper et al., 2020; Zhang H. et al., 2020; Zhang Y. et al., 2020). Therefore, we further investigated the relationship between FANCD2 expression and tumor immunity in LUAD.

We found that FANCD2 was closely associated with immunomodulating factors. A high level of FANCD2 expression upregulated the immune inhibitor expression of CTLA4, PDCD1, and LAG3 while downregulating immunostimulators, such as IL6R, TMEM173, TNFSF13, CD40LG, and HHLA2, which indicates that FANCD2 contributes to tumor immune escape by modulating the immunosuppressive microenvironment. This finding is consistent with previous studies (Hong et al., 2021; Yang et al., 2021). Moreover, higher FANCD2 expression led to a remarkable reduction in the infiltration of CD8^+^ T cells, NK cells, and DC cells, but it also recruited Th2 cells, and it is closely connected to the corresponding marker genes of tumor infiltrates immune cells (TIICs) based on the ssGSEA, CIBERSORT, and GEPIA databases. These results validate the role of FANCD2 in the LUAD immune system.

CD8^+^ T cells, as preferred cancer-targeting immunotherapy cells, through exocytosis and the release of perforin-granzyme and activation of caspases *via* the release of cytochrome c in cancer cells, contribute to tumor cell apoptosis (Farhood et al., 2019). NK cells are the first line of defense against tumors, and they not only release perforin and granzymes but also excrete various cytokines (IFN-γ, TNF), chemokines (IL10), or growth factors (GM-CSF) to play a crucial role in antitumor effects and antiviral infection. Notably, IFN-γ enhances the function of antigen-presenting cells, inhibits angiogenesis, induces Th1 cells, and promotes M1 macrophage polarization, which remarkably increases the effect of immune surveillance and immune elimination in the TME (Morvan and Lanier, 2016). In addition, CD141^+^ DCs express lymphotoxin beta transcripts to contribute to lymphocyte recruitment, the priming and proliferation of cytotoxic T cells, and CD1C^+^ subpopulations of DCs that promote the maintenance of immune memory (Nizzoli et al., 2016; Lavin et al., 2017). In contrast, Th2 cells secrete anti-inflammatory factors such as IL-4, IL-5, and IL-10 to weaken the anti-tumor immune response. GATA3, as the genetic marker of Th2 cells, was positively correlated with the FANCD2 level, and this not only promotes Th2 differentiation but also inhibits Th1 differentiation (Yagi et al., 2011). Hence, the Th2 shift in the TME is considered to promote tumor relapse, metastasis, and poor prognosis (Liu et al., 2019).

TAMs participate in angiogenesis and lymphangiogenesis, contributing to the progression of NSCLC (Hwang et al., 2020). However, this study showed that the FANCD2 expression level had no significant association with the infiltration of TAMs, but it could modulate the expression of CD80 and CCR5 to enhance the immunosuppressive function of TAMs. A previous study suggested that CD80 binds to the CTLA-4 receptor, inhibiting T-cell activation (Chikuma, 2017). TAMs could independently stimulate tumor cell growth and migration *via* the CCL5/CCR1/CCR5 axis (Pham et al., 2020). These findings revealed that FANCD2 plays a crucial role in recruiting different TIICs and regulating anti-tumor immunity.

In addition, Treg cell markers, such as FOXP3 and CD25, which have a crucial function in suppressing the antitumor immune response (Litwin et al., 2021), were strongly correlated with FANCD2 expression. Several studies have documented that live tumor-infiltrating Tregs are related to poor prognosis in NSCLC patients (Shimizu et al., 2010). However, apoptotic Treg cells also mediate immunosuppression *via* the adenosine and A2A pathways (Maj et al., 2017). Intriguingly, Treg cells that undergo ferroptosis caused by GPX4 deficiency potentiate antitumor immunity, characterized by high ratios of cytotoxic CD8^+^ T cells to CD4^+^ T cells in the TME (Xu et al., 2021). Therefore, inducing cell ferroptosis of Treg cells is also an antitumor treatment.

Based on the results of previous studies, there is a synergism between ferroptosis and immunomodulation (Hong et al., 2021; Xu et al., 2021; Yang et al., 2021). In TME, macrophages can be converted from M2 to M1, making more H_2_O_2_ available in the Fenton reaction, resulting in the ferroptosis of tumor cells (Zanganeh et al., 2016). An additional study demonstrated that activated CD8^+^T cells release IFN-γ to restrain system xc-uptake cystine, promoting tumor cell lipid peroxidation and subsequently contributing to ferroptosis (Wang et al., 2019). When tumor cells undergo ferroptosis, tumor antigens are released, which creates an immunogenic TME, thus enhancing the response to immunomodulation (Zhang F. et al., 2019).

Given the function of FANCD2 in ferroptosis regulation and TME and the infiltration level of TIICs, FANCD2 is a crucial molecule for synergetic ferroptosis-induction treatment and immunotherapy. Therefore, FANCD2 might be a powerful predictor of patient outcomes, and its expression level is a potential novel standard to select treatment options for Patients with LUAD clinically. Patients with a high level of FANCD2 are more suitable for ferroptosis-induction treatment and/or immunotherapy.

This study explored the predictive value of FANCD2 and uncovered a potential mechanism of activity in LUAD tumorigenesis. However, it has several limitations. First, this study was not a prospective study, and all data analyzed were obtained from public databases. Second, due to the inability to receive more detailed patient information, the baseline of the survival curves is unadjusted, and the results might be biased. Third, the functions of FANCD2 in ferroptosis and tumor immunity, as well as their mechanism, have not been clarified *in vitro or in vivo*. Clearly, clinical studies are needed to validate its prognostic value, and more in-depth experimental studies are required to reveal the mechanisms.

## Conclusions

This study systematically analyzed the role of FANCD2 in tumor progression, prognosis, and therapy for patients with LUAD. These results demonstrated that upregulated FANCD2 contributes to immune escape and was associated with worse outcomes for Patients with LUAD. It might be associated with FANCD2 participating in maintaining a stable tumor cell genome, protecting cells from ferroptosis, and constructing an immunosuppressive microenvironment. Furthermore, FANCD2 recruits immunosuppressive cells into the TME and regulates the expression of corresponding immune markers to weaken the anti-tumor immune response (Figure 10). Hence, FANCD2 is a biomarker for predicting human LUAD prognoses and may be a novel potential bio-target for identifying patients who may benefit from ferroptosis-induction treatment and/or immunotherapy.

## Data Availability

The datasets presented in this study can be found in online repositories. The names of the repository/repositories and accession number(s) can be found in the article/Supplementary Materials.

## Fundings

The present study was supported by the National Natural Science Foundation of China (grant No. 82004281), the China Postdoctoral Science Foundation (grant No. 2021T140427 and 2021M691986), and the Development Plan of Shandong Medical and Health Technology (grant No. 2019WS581).
