# Supplementary material for: Upregulation of Ferroptosis-Related Fanconi Anemia Group D2 is a Poor Prognostic Factor and an Indicator of Tumor Immune Cell Infiltration in Lung Adenocarcinoma
Source: Front Genet. 2022 May 11;13:825685. doi: 10.3389/fgene.2022.825685 (PMC9130730; doi:10.3389/fgene.2022.825685)
Supplement: Supplementary file 3 [file Table3.DOCX]

Call:

"coxph(formula = Surv(time = time, event = event) ~ Age + Gender + T.stage + N.stage, data= data)"

n= 362, number of events= 145

(164 observations deleted due to missingness)

coef exp(coef) se(coef) z Pr(>|z|)

T.stage=T1&T2 NA NA NA NA NA

T.stage=T3&T4 0.6338 1.8848 0.2381 2.662 0.007766

N.stage=N0 NA NA NA NA NA

N.stage=N1&N2&N3 0.6984 2.0105 0.1988 3.514 0.000442

M.stage=M0 NA NA NA NA NA

M.stage=M1 0.1935 1.2134 0.3274 0.591 0.554655

Pathologic.stage=Stage I&Stage II NA NA NA NA NA

Pathologic.stage=Stage III&Stage IV 0.3438 1.4103 0.2486 1.383 0.166719

FANCD2=Low NA NA NA NA NA

FANCD2=High 0.4015 1.4941 0.1727 2.325 0.020086

T.stage=T1&T2

T.stage=T3&T4 **

N.stage=N0

N.stage=N1&N2&N3 ***

M.stage=M0

M.stage=M1

Pathologic.stage=Stage I&Stage II

Pathologic.stage=Stage III&Stage IV

FANCD2=Low

FANCD2=High *

---

Signif. codes: 0 ‘***’ 0.001 ‘**’ 0.01 ‘*’ 0.05 ‘.’ 0.1 ‘ ’ 1

exp(coef) exp(-coef) lower .95 upper .95

T.stage=T1&T2 NA NA NA NA

T.stage=T3&T4 1.885 0.5306 1.1820 3.006

N.stage=N0 NA NA NA NA

N.stage=N1&N2&N3 2.011 0.4974 1.3618 2.968

M.stage=M0 NA NA NA NA

M.stage=M1 1.213 0.8241 0.6387 2.305

Pathologic.stage=Stage I&Stage II NA NA NA NA

Pathologic.stage=Stage III&Stage IV 1.410 0.7091 0.8663 2.296

FANCD2=Low NA NA NA NA

FANCD2=High 1.494 0.6693 1.0650 2.096

Concordance= 0.697 (se = 0.024 )

Likelihood ratio test= 54.78 on 5 df, p=1e-10

Wald test = 60.1 on 5 df, p=1e-11

Score (logrank) test = 65.22 on 5 df, p=1e-12
